# Landscape of clinical trial activity focusing on Indigenous health in Australia: an overview using clinical trial registry data from 2008-2018

**DOI:** 10.1186/s12889-022-13338-y

**Published:** 2022-05-14

**Authors:** Ge Xu, Danai Modi, Kylie E. Hunter, Lisa M. Askie, Lisa M. Jamieson, Alex Brown, Anna Lene Seidler

**Affiliations:** 1 NHMRC Clinical Trials Centre, the University of Sydney, Camperdown, NSW 2050 Australia; 2 Australian Research Centre for Population Oral Health, The University of Adelaide, Adelaide, Australia; 3 South Australian Health and Medical Research Institute, Adelaide, Australia

**Keywords:** Indigenous health, Clinical trial registration, Burden of disease, Australia, Population health, Research prioritisation, Minority health, Underserved

## Abstract

**Background:**

Aboriginal and Torres Strait Islander peoples (hereafter respectfully referred to as Indigenous Australians) represent about 3% of the total Australian population. Major health disparities exist between Indigenous and Non-Indigenous Australians. To address this, it is vital to understand key health priorities and knowledge gaps in the current landscape of clinical trial activity focusing on Indigenous health in Australia.

**Methods:**

Australian-based clinical trials registered on the Australian New Zealand Clinical Trials Registry or ClinicalTrials.gov from 2008 to 2018 were analysed. Australian clinical trials with and without a focus on Indigenous health were compared in terms of total numbers, participant size, conditions studied, design, intervention type and funding source.

**Results:**

Of the 9206 clinical trials included, 139 (1.5%) focused on Indigenous health, with no proportional increase in Indigenous trials over the decade (*p* = 0.30). Top conditions studied in Indigenous-focused trials were mental health (*n* = 35, 28%), cardiovascular disease (*n* = 20, 20%) and infection (*n* = 16, 16%). Compared to General Australian trials, Indigenous-focused trials more frequently studied ear conditions (OR 20.26, 95% CI 10.32–37.02, *p* < 0.001), infection (OR 3.11, 95% CI 1.88–4.85, *p* < 0.001) and reproductive health (OR 2.59, 95% CI 1.50–4.15, *p* < 0.001), and less of musculoskeletal conditions (OR 0.09, 95% CI 0.00–0.37, *p* < 0.001), anaesthesiology (OR 0.16, 95% CI 0.01–0.69, *p* = 0.021) and surgery (OR 0.17, 95% CI 0.01–0.73, *p* = 0.027). For intervention types, Indigenous trials focused more on prevention (*n* = 48, 36%) and screening (*n* = 18, 13%). They were far less involved in treatment (*n* = 72, 52%) as an intervention than General Australian trials (*n* = 6785, 75%), and were less likely to be blinded (*n* = 48, 35% vs *n* = 4273, 47%) or have industry funding (*n* = 9, 7% vs 1587, 17%).

**Conclusions:**

Trials with an Indigenous focus differed from General Australian trials in the conditions studied, design and funding source. The presented findings may inform research prioritisation and alleviate the substantial burden of disease for Indigenous population.

**Supplementary Information:**

The online version contains supplementary material available at 10.1186/s12889-022-13338-y.

## Introduction

High quality research that addresses health priority areas in culturally appropriate ways is needed to improve health outcomes, whilst taking into consideration the socioeconomic and environmental factors that make individuals susceptible to disease. Constituting 3.3% of the overall Australian population [1], the wellbeing of Aboriginal and Torres Strait Islander peoples (hereafter respectfully referred to as Indigenous Australians) has been an area of ongoing concern. This is due to persistent disparities in life expectancy and childhood mortality despite initiatives such as ‘Closing the Gap’ (2008) and ‘Closing the Gap Refresh’ (2018) [2]. Given that substantial funding has been directed towards Indigenous healthcare [3], a better understanding of Indigenous health-related research activity in relation to other Australian research activity is required to both highlight and address persisting inequities. Earlier studies focusing on Indigenous health have been criticised for their lack of impact on health outcomes and priorities [4, 5], and were conducted in isolation without comparison to other Australian studies.

The Australian New Zealand Clinical Trials Registry (ANZCTR) is one of 17 online clinical trial registries recognised as a Primary Registry within the World Health Organization’s International Clinical Trial Registry Network. Since its inception in 2005, the ANZCTR now displays over 95% of all clinical trials recruited in Australia, including those registered on ClinicalTrials.gov (CTgov) [6]. As trial registration is now mandated by the International Committee of Medical Journal Editors [7], the Declaration of Helsinki and Australian National Statement on Ethical Conduct in Human Research [8, 9], registered studies provide a reliable representation of overall trial activity.

The aim of this study was thus to use clinical trial registry data to examine the characteristics of interventional trials conducted in Australia focusing on Indigenous health and comparing these to Australian trials without Indigenous focus. Our secondary objective was to assess how well Indigenous trial activity corresponded to their relative burden of disease, thereby providing information for future research prioritisation.

## Methods

### Study design and included studies

We extracted all interventional studies registered on ANZCTR or CTgov from 1 November 2008 to 31 October 2018 that listed Australia as the only country of recruitment. Publicly available data on ANZCTR.org.au and Clinicaltrials.gov were used to perform this study, where the combined and cleaned dataset could be supplied upon request. Trials were included based on their date of registration as commencement or completion date may not be reported. Multi-national trials were excluded due to concerns that they would have less proportional representation of Indigenous participants from Australia. The final set of sampled trials was termed ‘All-Australian trials’. Within this sample, we searched electronically for a subset of trials with an Indigenous focus, which was distinguished by a specific emphasis on Indigenous health, involving either a high percentage of Indigenous participants or Indigenous service providers, or with dedicated subgroup analyses for Indigenous Australians. We included trials that had terms such as ‘Indigenous’, ‘Aboriginal’ or ‘Torres Strait’ in the trial registration record’s ‘Public Title’, ‘Inclusion Criteria’, ‘Brief Summary’, ‘Description of intervention(s) / procedure’ and ‘Ethics committee name’ fields. To validate our scope, clan names from the Australian Standard Classification of Cultural and Ethnic Groups were searched in the sampled All-Australian trials [10]. Eligibility of the included Indigenous-focused trials were then assessed independently by two reviewers with high agreement (kappa = 0.88, 96% agreement). The extracted subset of trials was termed ‘Indigenous-Australian trials’ and the remaining subset was termed ‘General Australian trials’. To test the reliability of our Indigenous trial search strategy, 200 trials were randomly selected from all included trials and manually screened for eligibility in the Indigenous trial subset. No additional Indigenous-Australian trials were identified via manual screening that had not already been captured via our electronic search.

### Measures

Trials in the Indigenous-Australian and General-Australian groups were compared in terms of numbers and characteristics, see Table 1. For our secondary objective, we compared the conditions studied in Indigenous-Australian trials against the top ten burden of disease conditions for Indigenous Australians using Australian Institute of Health and Welfare (AIHW) data [11]. Burden of disease for Indigenous Australians was measured by totalling Disability Adjusted Life Years (DALYs; the number of years lived with or lost due to a certain disease or injury) for each disease.

**Table 1:** Terms and definitions of trial characteristics analysed in the report^a^

Term	Definition
Sample size	Target sample size was used as a proxy for actual sample size if this metric was unavailable
Allocation	Whether a trial was randomised or non-randomised
Masking	Whether a trial was open or blinded
Intervention type	Categorised as diagnosis/prognosis, early detection/screening, prevention, treatment (surgery), treatment (devices), treatment (drugs), treatment (other), rehabilitation, lifestyle, behaviour, other interventions. Treatment: any encompasses treatment in surgery, devices, drugs and/or other. Each trial can select up to three intervention codes. Diagnosis / prognosis: study designed to evaluate one or more tests aimed at identifying a disease or health condition, or determining a patient’s prognosis. Early detection / screening: study that involves the systematic examination of a group of participants, in order to separate well persons from those who have an undiagnosed pathologic condition or who are at high risk. It could also refer to the initial evaluation of an individual, intended to determine suitability for a particular treatment modality or to detect specific markers or characteristics that may require further investigation. Prevention: study designed to assess one or more interventions aimed at preventing the development of a specific disease or health condition. Treatment: drugs: study designed to assess the effect(s) of one or more chemical or biological agents including vaccines. Treatment: surgery: study designed to assess the effect(s) of one or more manual or operative surgical techniques, whether in the fields of cosmetic, elective, experimental, plastic, or replacement surgery (performed to diagnose, treat, or prevent disease or other abnormal conditions). Treatment: devices: study designed to evaluate the use of any physical item used in medical treatment whether it be an instrument, piece of equipment, machine, apparatus, appliance, material or other article, and whether it is used alone or in combination with the intention of preventing, diagnosing, treating, and curing a disease or condition. Examples include: artificial limbs, contact lenses, ventilators, catheters, implants, vibration therapy machines. Treatment: other: studies that do not fall under the broad definitions of drug, surgical, or device trials. Examples include interventions such as exercise, physiotherapy, cognitive therapy, special diets, herbal medicines, web-based treatments, motivational classes, music therapy, stem cell interventions. Rehabilitation: studies designed to evaluate one or more interventions which aim to restore the physical or mental health, function and quality of life in participants who have had or are currently suffering from an illness or injury. Rehabilitation may be performed through physical therapy (e.g. physiotherapy, chiropractic) and/or education (e.g. diet and exercise advice/ counselling). Lifestyle: studies designed to investigate the effect of interventions which relate to a way of life or style of living. Interventions may aim to alter the attitudes, habits and values of a person or group, and how these participants cope with their physical, psychological, social, and economic environments on a day-to-day basis. Examples include diet and nutrition plans, exercise or physical activity programs, quit smoking programs. Behaviour: studies designed to assess the effect of interventions which aim to elicit or modify mental or physical actions, responses or conduct in a person or group. Examples of behavioural interventions include cognitive behavioural therapy, exercise behaviour interventions, and breast feeding behavioural interventions. Other interventions: studies that do not fit under any of the above categories. This should only be selected when no other options are adequate. Examples include prayer, singing, driving.
Primary sponsor	The individual, organisation, group or other legal person taking on responsibility for securing the arrangements to initiate and/or manage a study (including government body, hospital, university, commercial/industry sector, charities/societies/foundations, other collaborative groups, individual or other)
Funding	Main source of monetary, material or infrastructure support for the study (including government body, hospital, university, commercial sector/industry, charities/societies/foundations, other collaborative groups or individuals)
Industry involvement	Any evidence of industry involvement as primary sponsor, secondary sponsor, collaborator or funding source
Health conditions	Registrants can select up to ten per study, coded from Level 1 condition categories developed by UK Clinical Research Collaboration [12]. These are alternative and complementary medicine, anaesthesiology, blood, cancer, cardiovascular, diet and nutrition, ear, emergency medicine, eye, infection, inflammatory and immune system, injuries and accidents, human genetics and inherited disorders, mental health, metabolic and endocrine, musculoskeletal, neurological, oral and gastrointestinal, physical medicine/rehabilitation, renal and urogenital, reproductive health and childbirth, respiratory, skin, surgery, stroke and other. Public health was excluded as a health condition.

[12]

^a^ Refer to article for further variable definitions: Australian New Zealand Clinical Trials Registry, Data Field Definitions. 2019, https://www.anzctr.org.au/docs/ANZCTR%20Data%20field%20explanation.pdf?t=519 (March 2022, date last accessed)

### Analysis

Data were analysed using the open-source software R 3.5.1 [13]. Key trial characteristics were compared between Indigenous trials and General trials by calculating proportions per category for binary and categorical measures, and medians with interquartile range for continuous measures. Within each category of comparison, percentage calculations were adjusted by the total number of trials, not the number of entries for conditions as each trial could list up to fourteen condition codes and up to three intervention codes. We derived odds ratios (OR) with 95% confidence intervals (CI) in a logistic regression analysis, *p*-values for categorical comparisons using χ^2^ test and for nonparametric binary comparisons via Mann-Whitney U test. Co-variates were not adjusted since our aim was to provide a descriptive overview of Indigenous-Australian trials. The study methodology was designed in consultation with our Indigenous researcher and co-author. Ethical approval was not required as all trial data were publicly available and no human participants were recruited.

## Results

**Fig. 1:**
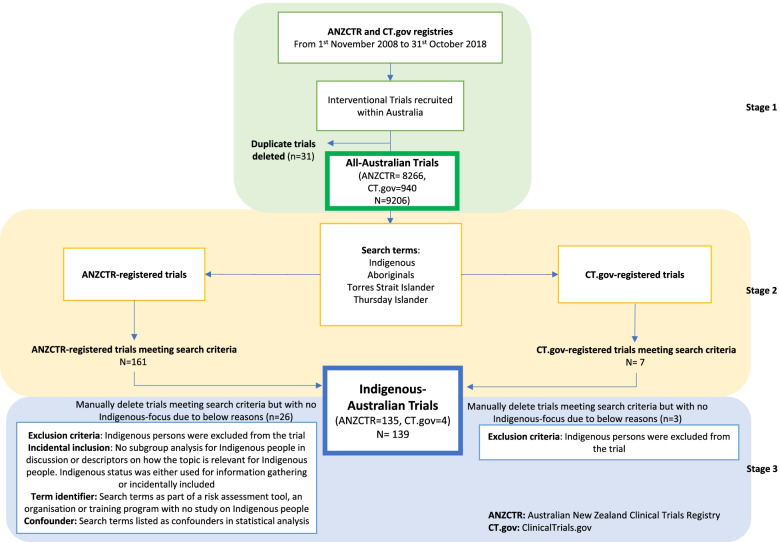
Selection process for Indigenous-Australian trials

The selection process for Indigenous-Australian trials is presented in Fig. 1. We identified 139 trials from 9206 All-Australian trials that were focused on Indigenous health (ANZCTR: 135; CTgov: 4). The remaining 9067 trials were termed General Australian (ANZCTR: 8131; CTgov: 936). An overview of results is provided in Table 2.

**Table 2:** Characteristics of Indigenous-Australian trials compared to general Australian trials

	Indigenous Australian trials n (%)	General Australian trials n (%)
Total number of trials	139	9067
Size		
Total number of participants	155,694	2,561,337^a^
Median sample size	250 *(IQR* 100–535)	60 (*IQR*30–140)
Public Health Involvement		
Public Health-related	59 (42%)	958 (11%)
Non-public health related	80 (58%)	8109 (89%)
Allocation		
Randomised	97 (70%)	6666 (74%)
Non-randomised	41 (30%)	2148 (24%)
Missing	1 (0%)	253 (3%)
Masking		
Blinded	48 (35%)	4273 (47%)
Open	76 (55%)	3935 (43%)
Missing	15 (10%)	859 (9%)
Intervention types^b^		
Treatment: any	72 (52%)	6785 (75%)
Prevention	48 (36%)	1343 (15%)
Behaviour	41 (30%)	1542 (17%)
Treatment: Other	35 (26%)	2465 (27%)
Lifestyle	26 (19%)	1016 (11%)
Treatment: Drugs	23 (17%)	2689 (30%)
Early detection / Screening	18 (13%)	298 (3%)
Other interventions	15 (11%)	618 (7%)
Treatment: Devices	7 (5%)	1270 (14%)
Treatment: Surgery	7 (5%)	361 (4%)
Rehabilitation	5 (4%)	728 (8%)
Diagnosis / Prognosis	3 (2%)	289 (3%)
Not applicable	0 (0%)	3 (0%)
Primary Sponsor^c^		
University	72 (53%)	2699 (33%)
Individual	16 (12%)	1882 (23%)
Government body	14 (10%)	315 (4%)
Other	13 (10%)	249 (3%)
Charities/Societies/Foundations	8 (6%)	272 (3%)
Other Collaborative groups	6 (4%)	227 (3%)
Hospital	3 (2%)	1619 (20%)
Commercial sector/Industry	3 (2%)	868 (11%)
Funding^d^		
Government body	107 (79%)	1930 (21%)
Charities/Societies/Foundations	19 (14%)	1542 (17%)
University	11 (8%)	1678 (18%)
Commercial sector/Industry	9 (7%)	1587 (17%)
Other Collaborative groups	7 (5%)	295 (3%)
Hospital	6 (4%)	1238 (13%)
Other	3 (2%)	241 (3%)
Self-funded/Unfunded	2 (1%)	759 (8%)

^a^ Two outliers were eliminated, each with participant size > 100,000 to avoid skewing of results and minimise misinterpretation of the mean recruitment size between Indigenous and General Australian trials

^b^ Percentage calculations were adjusted by the total number of trials, not the number of entries for intervention as each trial could list up to three intervention codes

^c^ Used ANZCTR data only, as CTgov had no data field for sponsorship

^d^ Used ANZCTR data only, as CTgov had fewer categories that could skew results. Each study could list up to 20 entries

**Fig. 2:**
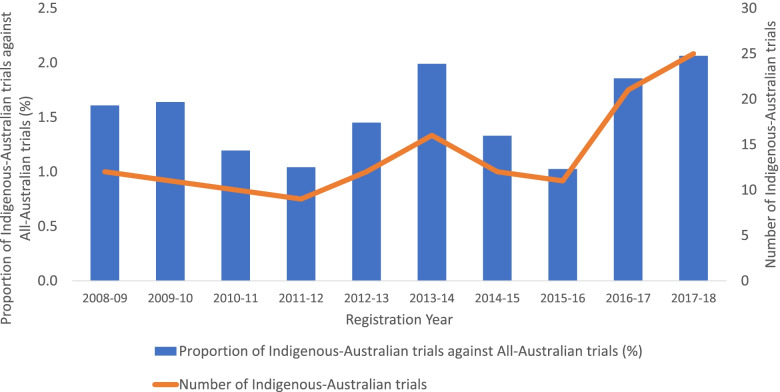
Percentage of Indigenous-Australian trials as a proportion of All-Australian trials (left) and absolute number of Indigenous-Australian trials (right) per registration year from 2008 to 2018

Over the ten-year study period, the absolute number of Indigenous-Australian trials increased from 12 in 2008–09 to 25 in 2017–18 (see Fig. 2). There was no significant increase in the proportion of Indigenous-Australian trials per year when compared against General Australian trials (χ^2^ (df) = 8.11 (9), *p* = 0.52) (Fig. 2). The total participant sample size for Indigenous-Australian trials was 155,694, which constituted 5.73% of the recruitment to the corresponding All-Australian trials (2,717,031) in the ten-year period. There was also no significant increase in the participant sample size of Indigenous trials when examined in proportion to All-Australian trials (see Supplementary Fig. 1). The median participant sample size of Indigenous-Australian trials (*n* = 250, *IQR* 100–535) was considerably larger than for General Australian trials (*n* = 60, *IQR*30–140) (Mann-Whitney *U* = 297,250, *p* < 0.001). There was no clear trend in median sample size over time for Indigenous-Australian or General Australian trials (post-hoc analysis, Supplementary Table 1). Indigenous-Australian trials were more likely to list public health as an area of study (59/139, 42%) compared to other Australian trials (958/9067, 11%) (OR 6.24, 95% CI 4.41–8.78).

**Fig. 3:**
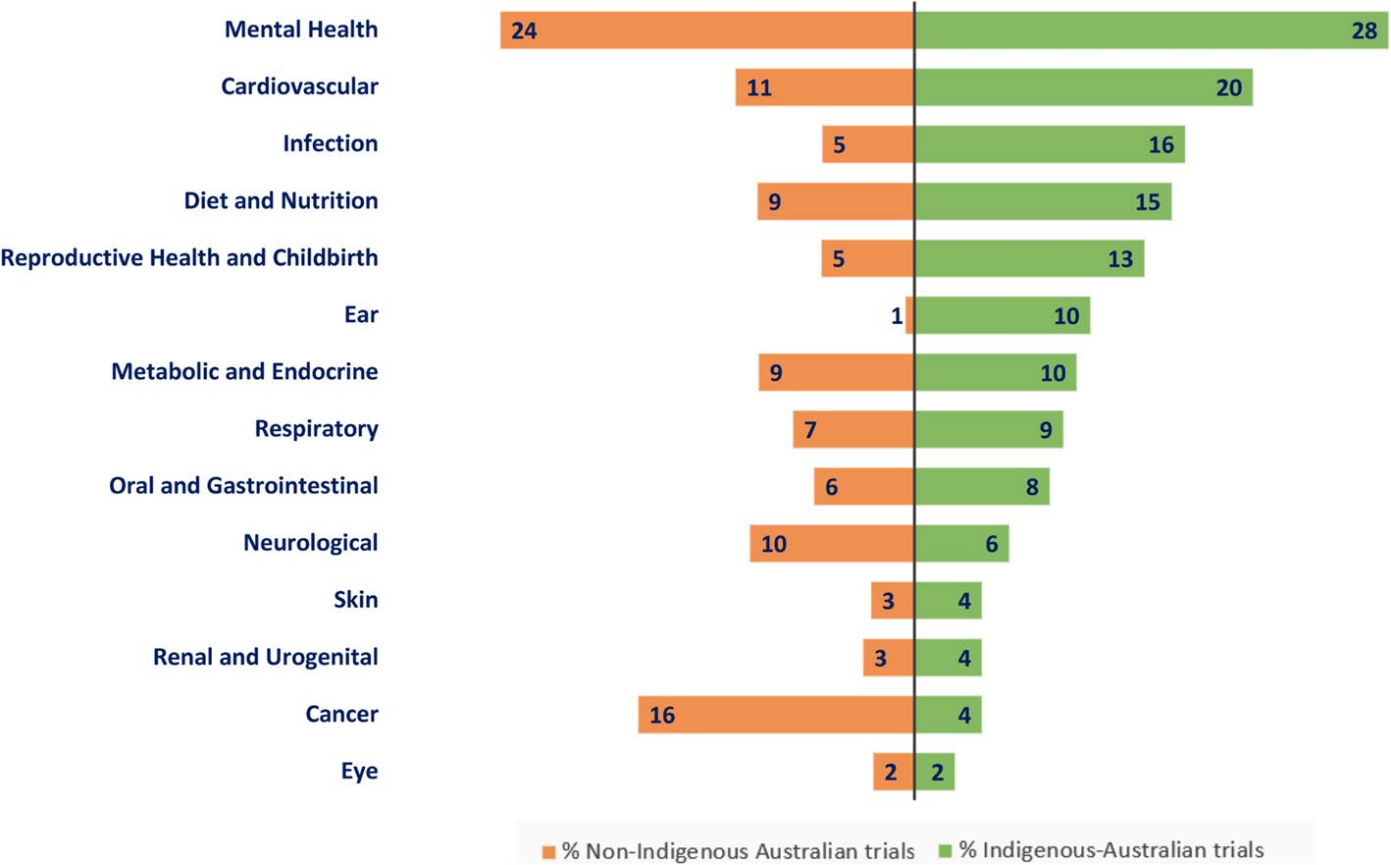
Top 14 conditions studied in Indigenous-Australian trials compared to General- Australian trials registered 2008–2018. Numbers within the bars are the percentage of trials in that category. Note that multiple conditions may be selected per trial therefore the percentages do not add to 100

**Fig. 4:**
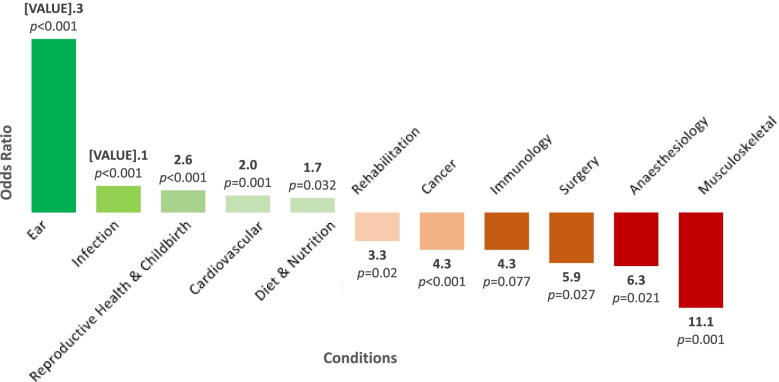
Odds ratios of conditions studied in Indigenous-Australian trials, compared to General Australian trials, 2008–2018

Allowing up to 14 registered conditions, the median and average number of health conditions registered per Indigenous-Australian trial were 1 (*IQR*1.00–2.00) and 1.62 (*SD*0.81) respectively, similar to General Australian trials where median was 1 (*SD*0.80) and mean was 1.60 (*IQR*1.00–2.00). The most frequently listed health conditions studied in Indigenous-Australian trials were mental health (28%), cardiovascular disease (20%) and infection (16%), compared to other Australian trials which were mental health (24%), cancer (16%) and cardiovascular disease (11%). The top 14 most frequently studied conditions for Indigenous and General Australian trials are shown in Fig. 3. Between 2008 and 2018, Indigenous-Australian trials were more likely than General Australian trials to study ear conditions (OR 20.26, 95% CI 10.32–37.02, *p* < 0.001), infection (OR 3.11, 95% CI 1.88–4.85, *p* < 0.001) and reproductive health (OR 2.59, 95% CI 1.50–4.15, *p* < 0.001). They were less likely to focus on musculoskeletal conditions (OR 0.09, 95% CI 0.00–0.37, *p* < 0.001), anaesthesiology (OR 0.16, 95% CI 0.01–0.69, *p* = 0.021) and surgery (OR0.17, 95% CI 0.01–0.73, *p* = 0.027). Health conditions that were most and least commonly studied by Indigenous-Australian trials compared to other trials are displayed in Fig. 4.

Regarding the use of randomisation (Table 2), Indigenous-Australian and General Australian trials did not significantly differ: 70% (97/139) and 74% (6666/9067) were randomised respectively (OR 0.76, 95% CI 0.53–1.11, *p* = 0.148). Blinding was less common in Indigenous-Australian trials (35%, 48/124) compared to General Australian trials (47%, 4273/9067) (OR 0.58, 95% CI 0.40–0.83, *p* = 0.003).

The most common categories of interventions studied in Indigenous-Australian trials were prevention (48/139, 36%) and behavioural interventions (41/139, 30%) (Table 2). Compared to General Australian trials, Indigenous-Australian trials were significantly more likely to focus on screening (OR 3.57, 95% CI 2.10–5.70, *p* < 0.001), prevention (OR 2.24, 95% CI 1.61–3.08, *p* < 0.001) and behavioural interventions (OR 1.58, 95%CI 1.11–2.20, *p* = 0.009) and less likely to focus on rehabilitation (OR 0.38, 95% CI 0.13–0.83, *p* = 0.021) and treatment (OR 0.40, 95% CI 0.30–0.52, *p* < 0.001).

The most common sponsors of Indigenous-Australian trials were universities (*n* = 72, 53%), individuals (*n* = 16, 12%) and government bodies (*n* = 14, 10%) shown in Table 2. For funding, Indigenous-Australian trials had higher rate of government (OR 2.90, 95% CI 1.57–4.93, *p* < 0.001) and universities (OR 2.30, 95% CI 1.63–3.24, *p* < 0.001) support and less funding by hospitals (OR 0.10 95% CI 0.02–0.25, *p* < 0.001) and industry (OR 0.20, 95% CI 0.05–0.53, *p* = 0.002). Additionally, only 11.5% (*n* = 16) of Indigenous-Australian trials had some form of industry involvement (as either a sponsor, collaborator or funder) compared to 24.9% (*n* = 2255) of General Australian trials (OR 2.52, 95% CI 1.54–4.43, *p* < 0.001).

**Fig. 5:**
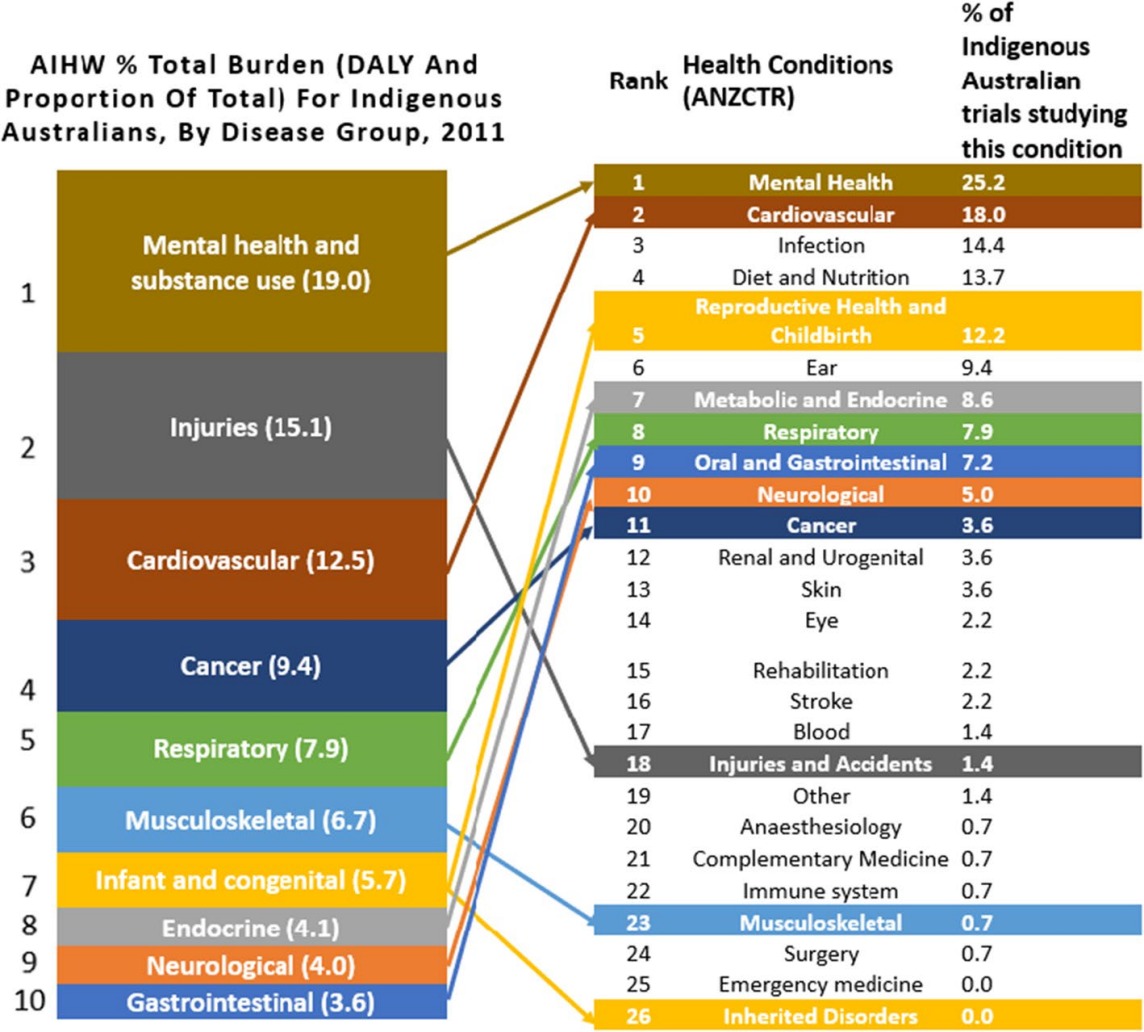
Comparison of the percentage of total burden of disease measured in DALY as a proportion of total from AIHW Burden of Disease study for Indigenous Australians (10) to percentage of Indigenous Australian trials studying various health conditions registered on ANZCTR and ClinicalTrials.gov

The AIHW data highlighted the conditions that contributed most to the burden of disease for Indigenous Australians (see left side of Fig. 5). Our analysis of the frequency that various conditions were studied in Indigenous-Australian trials shows that studied conditions do not necessarily align with research priorities. For example, whilst cardiovascular and mental health conditions were studied with high frequency in Indigenous-Australian trials between 2008 and 2018, which broadly reflects their contribution to the burden of disease, other conditions such as injuries and musculoskeletal disorders were studied less frequently than would be expected relative to their burden of disease. In terms of funding for research in the top ten burden of disease groups, government bodies were the most common funding sources, as shown in Supplementary Table 2 which divides funding into conditions from priority and non-priority areas. In comparison, industry funding for Indigenous-Australian trials was less common, and this affected conditions listed as priority areas (*n* = 12/162, 7.4% conditions funded from industry) and non-priority areas (*n* = 3/83, 3.6% conditions funded from industry). On examining the type of interventions used to address top ten burden of disease areas (see Supplementary Table 4), a high proportion of trials studying mental health conditions evaluated behavioural interventions (*n* = 22), whereas drug-related interventions were scarce, and studied mostly in cardiovascular (*n* = 5) research.

## Discussion

Our study examined registered trials focusing on the health of Indigenous Australians between 2008 and 2018 compared with other Australian-based trials. We found no significant proportional increase in the number or size of Indigenous-Australian trials, relative to General Australian trials, over the decade. Comparatively, Indigenous-Australian trials studied a higher proportion of public health-relevant topics with a significantly larger median participant sample size. They were also less likely to be blinded, more likely to study screening and preventive interventions, and were more commonly funded by universities and government compared to General Australian trials.

To our knowledge, this is the first study in Australia to use clinical trial registry data to provide an overview of Indigenous-focused clinical trial activity. Previously, reviews of Indigenous health research included only published trials with minimal comparison to other Australian trials [4, 5, 14, 15]. The advantage of utilising clinical trial registries is that they mandate information from each trial, therefore providing greater transparency, reduced publication bias and objective quantification of the data collected. Furthermore, our study had a rigorous search strategy for Indigenous-Australian trials, optimised with objective search terms and ratified by two independent reviewers in addition to an Indigenous researcher.

The absolute rise in the number of Indigenous trials from 2008 to 2018 can be seen as a continuation of the upward trend projected in previous reviews of published papers focusing on Indigenous health from 1995 to 2008 [14]. The lack of proportional growth was unexpected, given there had been an increase in the Australian National Health and Medical Research Council (NHMRC) funding for Indigenous health-related research from 2.9% in 2006, to 5% in 2008, and 6.3% in 2015–2016 [3, 16]. It is difficult to determine the reasons for this, since the ANZCTR does not collect data on the total funding or cost of a trial or the proportion of Indigenous Australians participating in General Australian trials. However, we postulate that this could be explained by increased participation of Indigenous people in General Australian trials, improved trial quality and potentially the increased cost of trials. For example, Indigenous-Australian trials had significantly larger median sample size with greater focus on public health, which may require more resources to conduct compared to other Australian trials – albeit at times smaller trials that require expensive drugs or equipment may cost more than large public health trials.

Our assessment of trial design has shown that blinding was less common amongst Indigenous-focused trials compared to other Australian trials. This may be due to increased emphasis on participatory-style of research for Indigenous Australians [17], also reflected in the study of Indigenous health in Canada and New Zealand [18, 19], which encourages a partnership between participants and researchers that makes masking difficult to implement. Alternatively, less blinding can reflect the types of interventions and conditions studied; since public health interventions, and studies of prevention, education or screening (which are more common in Indigenous-Australian trials) maybe harder to blind than a drug treatment trial. Our study demonstrates that the degree of randomisation was similar between Indigenous-Australian trials and other Australian trials. This challenges previous findings that randomisation is less common in Indigenous trials and potentially points toward better engagement with Indigenous communities through effective capacity exchange as promoted by the NHMRC Road Map 3 [3, 4].

In reference to the burden of disease analysis by AIHW [11], the numbers of registered trials studying mental health and cardiovascular disease broadly aligned with their burden of disease ranking. Mental health research amongst the Indigenous population has increased from less than 5% in the pre-2008 period to 25% in our study [14]. Other conditions such as injuries / accidents, cancer and musculoskeletal illness appear to have a larger discrepancy which may warrant more attention. For example, Indigenous people experience more head and neck cancers, later detection and reduced survival from all cancers compared with non-Indigenous people [20]. Similar cancer health disparities are seen in Indigenous populations in the United States and Canada [21, 22], suggesting a need for a better research framework. It should be noted that not all conditions with a high burden of disease require Indigenous-focused trials. Some diseases such as cardiovascular or musculoskeletal may not be population-specific and thus Indigenous Australians should be eligible for and encouraged to participate in them as they benefit both populations alike. However, certain conditions such as otitis media, rheumatic heart disease and untreated dental caries, may warrant targeted population study as they contribute significantly to Indigenous disease burden [3, 23, 24]. Additionally, it is important to note that the source of funding for trials studying areas with the greatest burden of disease for the Indigenous population was government bodies, which calls for further industry involvement in these priority areas.

### Limitations

Our study provides only a descriptive analysis of Indigenous-Australian trials in the decade from 2008 to 2018 and should thus not be used to draw causal inferences between Indigenous research and Indigenous health. Second, our study only captured registered trials and whilst registration rates on ANZCTR have been reported as being as high as 95% of all trials conducted [6], we may have potentially missed locally-conducted studies that were not registered. Additionally, we included trials based on their year of registration, which may vary from the year of commencement or completion. This would also affect the actual participant sample size which may be missing on initial entry, or different to the target participant sample size on registration. It is important to note that Indigenous Australians were/are likely eligible for most trials classified as General, with an unclear participation rate due to no explicit data. It was also beyond the scope of our study to critically appraise the research outcomes identified from each trial, and their overall impact on general health and health service usage. Last, our study had a limited capacity to reflect the social, environmental and cultural complexities of Indigenous health research using the traditional quantitative research framework we employed. For the Indigenous population, primary healthcare centres are at the forefront of disease prevention and management [25]. Our analysis compared research categories to health priority areas that were determined by DALYs which may be incongruent with the health priorities determined by local Indigenous communities or primary care physicians caring for Indigenous people.

## Conclusions

Research addressing areas of greatest disease burden may be one important way to improve life expectancy and reduce morbidity for Indigenous Australians. Our study has shown a steady growth in the absolute number, but not the proportion of trials with a focus on Indigenous health in Australia over the past 10 years. With growing focus on mental health and cardiovascular disease that are significant contributors to morbidity and mortality, further trials maybe needed in other health priority areas such as injuries/accidents and cancer for Indigenous Australians. The larger median sample size of Indigenous trials compared to other Australian trials, often with a focus on disease prevention rather than treatment interventions, may reflect a positive shift towards community-based research that addresses the social determinants of health affecting outcomes for Indigenous Australians. These findings could inform research prioritisation, which in turn may contribute to improved Indigenous wellbeing and life expectancy.

Considering future research, greater quantity of Indigenous-focussed trials can be achieved with increased funding from both public and industry sector. Better designed trials with high ethical standard can be realised from greater involvement of Indigenous authors, stakeholders, and health services. Additional trial analysis should examine the participation of minority population in mainstream trials to address the ongoing need for inclusivity of Indigenous Australians in studying health conditions non-specific to the population. Finally, as researchers continually address health priority areas, future research should also develop strategies that empower the Indigenous community so results can be reciprocated in engaging and culturally sensitive ways.

## Supplementary Information

**Additional file 1: Supplementary Table 1.** Comparison of the median sample size between Indigenous-Australian and General Australian trials based on registration year from 2008-2018. **Supplementary Table 2.** Types of funding displayed in absolute number and (percentage) for Indigenous-Australian trials registered from 2008-2018, for top 10 priority (and other) conditions as per Australian Institute of Health and Welfare (AIHW) % Total Burden for Indigenous Australians, by disease group 2011. (Note that one trial can study multiple conditions hence numbers do not reflect the number of trials). **Supplementary Table 3.** Participant Size in each respective year for All-Australian, General Australian and Indigenous Australian trials and Participant Size for Indigenous Australian Trials demonstrated as a proportion to All Australian Trials, 2008-2018. **Supplementary Table 4.** Types of intervention assigned for each health condition enlisted in Indigenous-Australian trials registered from 2008-2018, where included health conditions are from top 10 priority areas as per Australian Institute of Health and Welfare (AIHW) % Total Burden for Indigenous Australians, by disease group 2011. **Supplementary Figure 1.** Absolute sample size of Indigenous Australian Trials and as a proportion of All Australian Trials, 2008-2018.

## Data Availability

Publicly available data on ANZCTR.org.au and Clinicaltrials.gov were used to perform this study, where the combined and cleaned dataset can be supplied upon request to the corresponding author.

## Abbreviations

AIHW: Australian Institute of Health and Welfare
ANZCTR: Australia New Zealand Clinical Trials Registry
CI: Confidence interval
CTgov: ClinicalTrials.gov
DALYs: Disability adjusted life years
IQR: Interquartile range
NHMRC: National Health and Medical Research Council
OR: Odds ratios
SD: Standard deviation
TIA: Therapeutic Innovation Australia

## References

[1] Australian Institute of Health and Welfare. Profile of Indigenous Australians. Canberra: 2021. https://www.aihw.gov.au/reports/australias-welfare/profile-of-indigenous-australians. Accessed 6 Mar 2022.

[2] National Indigenous Australian Agency. About Closing the Gap. Australian Government; 2020. https://closingthegap.niaa.gov.au/about-closing-gap. Accessed 15 Apr 2020.

[3] National Health and Medical Research Council (2018). Road map 3: a strategic framework for improving the health of Aboriginal and Torres Strait islander people through research National Health and Medical Research Council, Australian Government.

[4] Kinchin I, Mccalman J, Bainbridge R, Tsey K, Lui FW (2017). Does indigenous health research have impact? A systematic review of reviews. Int J Equity Health.

[5] Morris P (1999). Randomised controlled trials addressing Australian Aboriginal health needs: a systematic review of the literature. J Paediatr Child Health.

[6] Tan AC, Jiang I, Askie L, Hunter K, Simes RJ, Seidler AL (2019). Prevalence of trial registration varies by study characteristics and risk of bias. J Clin Epidemiol.

[7] International Committee of Medical Journal Editors. Recommendations: Publishing and Editorial Issues. 2019. http://www.icmje.org/recommendations/browse/publishing-and-editorial-issues/clinical-trial-registration.html. Accessed 13 Aug 2019.

[8] World Medical Association. WMA Declaration Of Helsinki – Ethical Principles For Medical Research Involving Human Subjects. 2013. https://www.wma.net/policies-post/wma-declaration-of-helsinki-ethical-principles-for-medical-research-involving-human-subjects/. Accessed 10 Dec 2018.

[9] National Health and Medical Research Council (2007). The Australian Research Council, universities Australia: National Statement on ethical conduct in human research 2007 (updated 2018).

[10] Australian Bureau of Statistics. Australian standard classification of cultural and ethnic groups. Australian Government Canberra; 2011. http://www.abs.gov.au/AUSSTATS/abs@.nsf/DetailsPage/1249.02016?. Accessed 19 Dec 2018.

[11] Australian Institute of Health and Welfare (2016). Australian Burden of Disease Study: impact and causes of illness and death in Aboriginal and Torres Strait Islander people 2011. In: Australian Burden of Disease Study Series No: 62016.

[12] United Kingdom Clinical Research Collaboration. Health research classification system: Health categories. https://hrcsonline.net/health-categories/. Accessed 25 Mar 2020.

[13] R Development Core team (2018). R: a language and environment for statistical computing.

[14] Rumbold AR, Cunningham J, Purbrick B, Lewis JM (2013). Exploring productivity and collaboration in Australian indigenous health research, 1995–2008. Health Res Policy Syst.

[15] Azzopardi PS, Kennedy EC, Patton GC, Power R, Roseby RD, Sawyer SM, Brown AD (2013). The quality of health research for young indigenous Australians: systematic review. Med J Aust.

[16] Leon de la Barra S, Redman S, Eades S, Lonsdale C (2009). A decade of NHMRC people support expenditure in review: is support for indigenous health research increasing?. Med J Aust.

[17] National Health and Medical Research Council (2018). Ethical conduct in research with Aboriginal and Torres Strait islander peoples and communities: guidelines for researchers and stakeholders.

[18] Richmond CA, Cook C (2016). Creating conditions for Canadian Aboriginal health equity: the promise of healthy public policy. Public Health Rev.

[19] Reid P, Paine S-J, Curtis E, Jones R, Anderson A, Willing E, Harwood M (2017). Achieving health equity in Aotearoa: strengthening responsiveness to Māori in health research. N Z Med J.

[20] Australian Institute of Health and Welfare. Cancer in Aboriginal & Torres Strait Islander people of Australia. Canberra: 2011. https://www.aihw.gov.au/reports/cancer/cancer-in-indigenous-australians. Accessed 31 Aug 2020.

[21] Leon Guerrero RT, Palafox NA, Hattori-Uchima MP, Robinett HR, Vogel C-W (2020). Addressing Cancer health disparities in the Pacific peoples of Hawai ‘i, Guam, and the US associated Pacific Islands through Pacific-focused research capacity building. JCO Global Oncology.

[22] Letendre A, Garvey G, King A, King M, Crowshoe R, Bill L, Caron NR, Elias B (2020). Creating a Canadian indigenous research network against cancer to address indigenous cancer disparities. JCO Global Oncology.

[23] Christian B, Blinkhorn A (2012). A review of dental caries in Australian Aboriginal children: the health inequalities perspective. Rural Remote Health.

[24] Australian Institute of Health and Welfare (2018). Australia's health 2018: 6.3 indigenous child mortality and life expectancy.

[25] Jackson Pulver L, Haswell MR, Ring I, Waldon J, Clark W, Whetung V, Kinnon D, Graham C, Chino M, LaValley J (2010). Indigenous health: Australia, Canada, Aotearoa, New Zealand and the United States: laying claim to a future that embraces health for us all.

