# Supplementary material for: Landscape of clinical trial activity focusing on Indigenous health in Australia: an overview using clinical trial registry data from 2008-2018
Source: BMC Public Health. 2022 May 14;22:971. doi: 10.1186/s12889-022-13338-y (PMC9107126; doi:10.1186/s12889-022-13338-y)
Supplement: Supplementary file 1 — Additional file 1: Supplementary Table 1. Comparison of the median sample size between Indigenous-Australian and General Australian trials based on registration year from 2008-2018. Supplementary Table 2. Types of funding displayed in absolute number and (percentage) for Indigenous-Australian trials registered from 2008-2018, for top 10 priority (and other) conditions as per Australian Institute of Health and Welfare (AIHW) % Total Burden for Indigenous Australians, by disease group 2011. (Note that one trial can study multiple conditions hence numbers do not reflect the number of trials). Supplementary Table 3. Participant Size in each respective year for All-Australian, General Australian and Indigenous Australian trials and Participant Size for Indigenous Australian Trials demonstrated as a proportion to All Australian Trials, 2008-2018. Supplementary Table 4. Types of intervention assigned for each health condition enlisted in Indigenous-Australian trials registered from 2008-2018, where included health conditions are from top 10 priority areas as per Australian Institute of Health and Welfare (AIHW) % Total Burden for Indigenous Australians, by disease group 2011. Supplementary Figure 1. Absolute sample size of Indigenous Australian Trials and as a proportion of All Australian Trials, 2008-2018. [file 12889_2022_13338_MOESM1_ESM.docx]

# Title

Landscape of clinical trial activity focusing on Indigenous health in Australia: an overview using clinical trial registry data from 2008-2018

# Supplementary

**Supplementary Table 1.** Comparison of the median sample size between Indigenous-Australian and General Australian trials based on registration year from 2008-2018.

| Registration Year | Median sample size of Indigenous Australian trials (Interquartile range, number of trials) | Median sample size of General Australian Trials (Interquartile range, number of trials) |
| --- | --- | --- |
| 2008-09 | 430 (255-1000, n=12) | 68 (32-152, n=746) |
| 2009-10 | 200 (84-260, n=11) | 60 (30-140, n=668) |
| 2010-11 | 357 (166-1612, n=10) | 60 (30-150, n=836) |
| 2011-12 | 120 (70-250, n=9) | 51 (27-120, n=864) |
| 2012-13 | 342 (138-605, n=12) | 60 (28-145, n=825) |
| 2013-14 | 290 (81-470, n=16) | 59 (26-131, n=804) |
| 2014-15 | 251 (110-625, n=12) | 60 (28-132, n=901) |
| 2015-16 | 473 (250-930, n=11) | 60 (30-136, n=1073) |
| 2016-17 | 180 (91-466, n=21) | 60 (30-140, n=1130) |
| 2017-18 | 200 (100-400, n=25) | 60 (30-130, n=1212) |

| **Funding Groups** | **Conditions studied in Indigenous-Australian trials (top 10 priority areas as per AIHW), by funding group (%)** | | | | | | | | | | **Other Conditions studied (non-priority)** |
| --- | --- | --- | --- | --- | --- | --- | --- | --- | --- | --- | --- |
|  | Mental Health | Injuries  and Accidents | Cardio-vascular | Cancer | Respiratory | Musculo-skeletal | Infant and Congenital* | Metabolic and Endocrine | Neurological | Oral and Gastro-intestinal |  |
| Government body | 32 (64) | 2 (100) | 18 (69) | 3 (60) | 8 (62) | 1 (100) | 16 (46) | 7 (54) | 7 (78) | 8 (100) | 57 (69) |
| Charities/Societies  /Foundations | 6 (12) | 0 (0) | 4 (15) | 0 (0) | 3 (23) | 0 (0) | 2 (6) | 1 (8) | 2 (22) | 0 (0) | 5 (6) |
| University | 2 (4) | 0 (0) | 2 (8) | 2 (40) | 1 (8) | 0 (0) | 1 (3) | 1 (8) | 0 (0) | 0 (0) | 10 (12) |
| Commercial sector/Industry | 3 (6) | 0 (0) | 1 (4) | 0 (0) | 0 (0) | 0 (0) | 6 (17) | 2 (15) | 0 (0) | 0 (0) | 3 (4) |
| Hospital | 1 (2) | 0 (0) | 0 (0) | 0 (0) | 0 (0) | 0 (0) | 8 (23) | 1 (8) | 0 (0) | 0 (0) | 4 (5) |
| Other | 1 (2) | 0 (0) | 1 (4) | 0 (0) | 0 (0) | 0 (0) | 0 (0) | 0 (0) | 0 (0) | 0 (0) | 2 (2) |
| Other Collaborative groups | 4 (8) | 0 (0) | 0 (0) | 0 (0) | 1 (8) | 0 (0) | 2 (6) | 0 (0) | 0 (0) | 0 (0) | 1 (1) |
| Self-funded/Unfunded | 1 (2) | 0 (0) | 0 (0) | 0 (0) | 0 (0) | 0 (0) | 0 (0) | 1 (8) | 0 (0) | 0 (0) | 1 (1) |
| Total (%) | 50 (100) | 2 (100) | 26 (100) | 5 (100) | 13 (100) | 1 (100) | 35 (100) | 13 (100) | 9 (100) | 8 (100) | 83 (100) |

**Supplementary Table 2.** Types of funding displayed in absolute number and (percentage) for Indigenous-Australian trials registered from 2008-2018, for top 10 priority (and other) conditions as per Australian Institute of Health and Welfare (AIHW) % Total Burden for Indigenous Australians, by disease group 2011. (Note that one trial can study multiple conditions hence numbers do not reflect the number of trials)

* Infant and Congenital is the health condition term used in AIHW analysis, which includes both Reproductive Health and Childbirth as well as Inherited Disorders that are used to categorise conditions from the ANZCTR Database for Indigenous-Australian trials.

**Supplementary Table 3.** Participant Size in each respective year for All-Australian, General Australian and Indigenous Australian trials and Participant Size for Indigenous Australian Trials demonstrated as a proportion to All Australian Trials, 2008-2018

| Year | Total sample size (sum) for All-Australian Trials | Total sample size (sum) for General Australian Trials | Total sample size (sum) for Indigenous Australian Trials | Total sample size for Indigenous Australian Trials as a proportion to All Australian trials (%) |
| --- | --- | --- | --- | --- |
| 2008-09 | 269833 | 258333 | 11500 | 4.26 |
| 2009-10 | 123128 | 120681 | 2447 | 1.99 |
| 2010-11 | 388785 | 330457 | 58328 | 15.00 |
| 2011-12 | 167050 | 149338 | 17712 | 10.60 |
| 2012-13 | 275604 | 267521 | 8083 | 2.93 |
| 2013-14 | 210524 | 204358 | 6166 | 2.93 |
| 2014-15 | 307997 | 300136 | 7861 | 2.55 |
| 2015-16 | 262748 | 244898 | 17850 | 6.79 |
| 2016-17 | 619155 | 600469 | 18686 | 3.02 |
| 2017-18 | 373207 | 366146 | 7061 | 1.89 |

| **Intervention Type** | **Conditions studied from Indigenous-Australian trials (top 10 priority areas as per AIHW)** | | | | | | | | | |
| --- | --- | --- | --- | --- | --- | --- | --- | --- | --- | --- |
|  | Mental Health | Injuries and Accidents | Cardio-vascular | Cancer | Respiratory | Musculo-skeletal | Infant and Congenital | Metabolic and Endocrine | Neurological | Oral and Gastro-intestinal |
| Behaviour | 22 | 0 | 5 | 2 | 2 | 0 | 7 | 2 | 3 | 1 |
| Combination Product | 0 | 0 | 0 | 0 | 0 | 0 | 0 | 0 | 0 | 0 |
| Diagnosis / Prognosis | 0 | 0 | 1 | 0 | 0 | 0 | 0 | 0 | 0 | 0 |
| Early detection / Screening | 3 | 1 | 2 | 3 | 0 | 0 | 0 | 2 | 0 | 1 |
| Lifestyle | 10 | 0 | 5 | 1 | 0 | 0 | 3 | 1 | 0 | 1 |
| Other interventions | 0 | 0 | 2 | 0 | 1 | 1 | 1 | 2 | 2 | 1 |
| Prevention | 10 | 0 | 9 | 1 | 3 | 0 | 5 | 1 | 1 | 7 |
| Rehabilitation | 0 | 1 | 4 | 0 | 2 | 0 | 0 | 0 | 1 | 0 |
| Treatment: Devices | 4 | 0 | 0 | 0 | 0 | 0 | 0 | 0 | 0 | 0 |
| Treatment: Drugs | 4 | 0 | 5 | 0 | 3 | 0 | 1 | 2 | 1 | 2 |
| Treatment: Other | 12 | 1 | 6 | 0 | 2 | 0 | 10 | 4 | 0 | 5 |
| Treatment: Surgery | 0 | 0 | 1 | 0 | 0 | 0 | 0 | 1 | 0 | 2 |
| Not applicable | 0 | 0 | 0 | 0 | 0 | 0 | 0 | 0 | 0 | 0 |

**Supplementary Table 4.** Types of intervention assigned for each health condition enlisted in Indigenous-Australian trials registered from 2008-2018, where included health conditions are from top 10 priority areas as per Australian Institute of Health and Welfare (AIHW) % Total Burden for Indigenous Australians, by disease group 2011.

* Infant and Congenital is the health condition term used in AIHW analysis, which includes both Reproductive Health and Childbirth as well as Inherited Disorders that are used to categorise conditions from the ANZCTR Database for Indigenous-Australian trials.

**Supplementary Figure 1**. Absolute sample size of Indigenous Australian Trials and as a proportion of All Australian Trials, 2008-2018
